# Cybersecurity Awareness and Training (CAT) Framework for Remote Working Employees

**DOI:** 10.3390/s22228663

**Published:** 2022-11-09

**Authors:** Mohammad Hijji, Gulzar Alam

**Affiliations:** 1 Faculty of Computers & Information Technology, University of Tabuk, Tabuk 71491, Saudi Arabia; 2 School of Computing, Ulster University, Belfast BT15 1ED, Northern Ireland, UK

**Keywords:** artificial intelligence, cybersecurity, COVID-19, education, cybersecurity awareness, training framework

## Abstract

Currently, cybersecurity plays an essential role in computing and information technology due to its direct effect on organizations’ critical assets and information. Cybersecurity is applied using integrity, availability, and confidentiality to protect organizational assets and information from various malicious attacks and vulnerabilities. The COVID-19 pandemic has generated different cybersecurity issues and challenges for businesses as employees have become accustomed to working from home. Firms are speeding up their digital transformation, making cybersecurity the current main concern. For software and hardware systems protection, organizations tend to spend an excessive amount of money procuring intrusion detection systems, antivirus software, antispyware software, and encryption mechanisms. However, these solutions are not enough, and organizations continue to suffer security risks due to the escalating list of security vulnerabilities during the COVID-19 pandemic. There is a thriving need to provide a cybersecurity awareness and training framework for remote working employees. The main objective of this research is to propose a CAT framework for cybersecurity awareness and training that will help organizations to evaluate and measure their employees’ capability in the cybersecurity domain. The proposed CAT framework will assist different organizations in effectively and efficiently managing security-related issues and challenges to protect their assets and critical information. The developed CAT framework consists of three key levels and twenty-five core practices. Case studies are conducted to evaluate the usefulness of the CAT framework in cybersecurity-based organizational settings in a real-world environment. The case studies’ results showed that the proposed CAT framework can identify employees’ capability levels and help train them to effectively overcome the cybersecurity issues and challenges faced by the organizations.

## 1. Introduction

Cybersecurity performs an important role nowadays by protecting government data, business, industrial information, intellectual property, and personal information from hackers and cybercriminals. Extensive use of technology is directly proportional to the increase in cybercrime. The main objective of cybersecurity is, therefore, to protect information due to the enormous increase in cyber-attacks and to lessen the possibility of global and catastrophic consequences [1]. Researchers and practitioners are developing best practices to protect computer systems, devices, networks, and software systems from unauthorized access or cyber threats.

The COVID-19 pandemic has generated different challenges for businesses as employees have become accustomed to working from home. Firms are speeding up their digital transformation, making cybersecurity the current main concern [2]. The reputational, operational, legal, and compliance consequences may be substantial if cybersecurity threats are ignored. The upsurge in remote working demands more attention regarding cybersecurity because of the larger exposure to cyber-attacks, threats, and risks, with 47% of employees and individuals experiencing an attempted phishing scam whilst working at home. Cyber-attackers and hackers perceive the COVID-19 pandemic as an opportunity to step up their illegal behaviors by manipulating the vulnerability of employees working from home. Likewise, one more significant consequence is that the average cost of a data breach caused by remote working employees is USD 137,000 [3]. Hijji and Alam [4] conducted a comprehensive multi-vocal literature review on the increasing social-engineering-based cyber-attacks and threats during the COVID-19 pandemic. They mentioned attacked organization domains, the platform used for cyber-attacks, and the types of malware used. Most of the attacks are performed by using social-engineering-based tactics to manipulate workers into opening suspicious links sent via different social platforms. Currently, awareness and training are very important for updating employees regarding the recent kinds of cyber-attacks and threats and to protect their organizations from huge financial, personal, and reputation loss.

Sibillon et al. [5] proposed a cybersecurity awareness training model (CATRAM) for cybersecurity culture awareness to support training of various organizational employees. Their model was validated through a case study in Canada. Similarly, Rajamäki et al. [6] recommended a “Proactive Resilience Educational Framework (Proresilience EF)” to facilitate cybersecurity education and training in the healthcare field. Similarly, Alshaikh et al. [7] constructed “Information Security Education Training and Awareness (SETA)” To provide sustainable behavioral change regarding cybersecurity by adopting the behavior change wheel (BCW) framework. A detailed comparison of the other relevant cybersecurity frameworks/models is shown in Table 1. However, no such model exists to provide awareness to and to train online-working-based employees.

The main objective of this research is to develop a cybersecurity awareness and training framework to support organizations in enriching and training their employees to secure the information and overall system of the organization. We studied the existing empirical literature on different published cybersecurity frameworks and models with the inclusion of the structural concept of the well-known NIST “Building an information technology security awareness and training program” [8]. We developed CAT by introducing AI concepts consisting of machine learning, natural language processing, and expert systems to make our proposed model self-adaptive and smart.

This research study is organized as follows: Section 2 comprehensively explains the related work and motivation of cybersecurity and the existing frameworks/models. The research methodology is discussed in detail in Section 3. Similarly, Section 4 represents the proposed CAT framework. Section 5 includes the case studies and evaluation of the CAT framework in the real-world industrial environment, and Section 6 reviews the post-case-study evaluation and feedback. Finally, Section 7 presents the limitations of the research study, and Section 8 presents the conclusions and future work.

## 2. Motivation and Related Work

In recent times, academia and industry have been paying more attention to cybersecurity. ISO 9126 [9] described security as a sub-characteristic of software functionality; however, in the reviewed updated version (ISO 20510) [10], software security has been included as a main superiority feature, with sub-characteristics such as confidentiality, integrity, non-repudiation, accountability, and authenticity due to its significance. ISO 25010 defines software security as “the degree to which a product or system protects information and data so that persons or other products or systems have the degree of data access appropriate to their types and levels of authorization” [10]. Further, McGraw defined software security as “the ability of software to resist, tolerate, and recover from events that intentionally threaten its dependability” [11].

Similarly, numerous research papers have been proposed on the vulnerability classifications and taxonomies [12,13,14,15,16] due to growing complexities in software and hardware systems. Therefore, the possible scope of cybersecurity has also meaningfully grown. Woodhouse [17] proposed the Information Security Management System (ISMS) model for assessing the processes capability and maturity within an organization. ISMS defined nine process maturity levels, namely: “Subversive, Arrogant, Obstructive, Negligent, Functional, Technical, Operational, Managed and Strategic”. Likewise, Almuhammadi [18] developed the “Information Security Maturity Model (ISMM)” for the NIST cybersecurity framework with a curiosity to fill the gap of NIST-CSF (Framework for Critical Infrastructure). Moreover, Kassou and Kjiri [19] constructed the Service-Oriented Architecture Security Maturity Model (SOASMM) and established some security practices and standards to support companies in evaluating and normalizing their security according to their Service-Oriented Architecture (SOA). Correspondingly, White [20] presented a “Community Cyber Security Maturity Model (CCSMM)” that benefits a program developed by approving these three main mechanisms: (1) cybersecurity posture and maturity, (2) security posture improvements, and (3) common point of reference. In the same way, Thomson and Solms [21] proposed the Information Security Competence Maturity Model (ISCMM) for assessment and evaluation of information security levels.

Specifically, Ghazvini and Shukur [22] proposed a framework for the healthcare community by building guidelines regarding information security training and its methods of delivery. They evaluated their framework in the healthcare domain via a case study. Similarly, Aliyu et al. [23] developed a cybersecurity maturity framework to assess and evaluate the security and privacy regulations with optimal practice in higher educational institutes of the UK. Furthermore, Georgiadou et al. [24] presented a procedure for assessing the cybersecurity culture of an organization by considering the significance of human factors. Finally, Sibillon et al. [5] proposed a cybersecurity awareness training model (CATRAM) for cybersecurity culture awareness to support training of various organizational employees. Their proposed model was validated through a case study in Canada.

Hong et al. [25] measured the impact of education level of great societies to moderate the correlation in the middle of knowledge and attitude by using the knowledge–attitude–behavior (KAB) model. Sabillon [26] introduced a model to evaluate the application and support of the cybersecurity awareness training model (CATRAM), constructed based on multiple case studies in Canadian higher education organizations. They intended to provide training to various corporate spectators by covering the overall cyberthreat background. Similarly, Alghamdi [27] conducted a case study in Saudi Arabia to find out the effect of cybersecurity awareness on worker behavior with the temperance impact of cybersecurity policy awareness level. Likewise, Ungkap and Daengsi [28] proposed an approach to find out and assess the important factors that affect cybersecurity awareness of the internet users. They considered Thailand railway organization users to detect and evaluate the factors connected to cybersecurity awareness of the users successfully. Further, Daengsi et al. [29] performed comparative research work on cybersecurity awareness on phishing attacks amongst workers from various departments of an organization in Bangkok. They identified that the organization workers demonstrated inadequate performance towards fishing attacks due to their deficient awareness of cybersecurity. Moreover, Back et al. [30] examined the efficiency of home management methods on decreasing cyber-attacks and threat incidents during online conditions. They focused on the impact of phishing attacks conducted through emails and suspicious links. According to the literature reviews of the researchers, Corallo et al. [31] conducted a systematic literature review on cybersecurity awareness in the domain of industrial internet of things. Kennedy and Chiasson [32] performed a thorough systematic review containing scholarly publications and industry tools/software concerning cybersecurity awareness and education intended for online working users that were established in the previous 20 years. They discovered 119 software and tools, uncovered existing trends, evaluated their usage of appropriate instructional pattern standards, and examined the pragmatic proofs of the software and tools’ efficiency. As an outcome, they delivered an assessment specification list and recommended that a more organized and systematic methodology for design and appraisal of cybersecurity educational software and tools would be an advantageous step.

Table 1 shows some of the recent cybersecurity awareness and training frameworks/approaches/models with a name, objective, domain, and validation procedure, such as case study, survey, experiments, and interviews with experts in the cybersecurity field. The proposed frameworks/approaches/models in Table 1 were built for various domains, including healthcare, education, industry (organization), and business.

**Table 1:** The current cybersecurity awareness and training frameworks/approaches/models.

Year	Reference	Framework	Objective	Domain	Validation
2017	[22]	Information security awareness program	To provide training delivery method and guidelines	Healthcare	Case Study
2020	[23]	Cybersecurity Maturity AssessmentFramework	Developed cybersecurity assessment framework for Higher Education Institutions	Education	Case Study
2019	[33]	National Cyber Governance Awareness Policy Framework	To assess cybersecurity governance and awareness at the government, national, and law prosecution level	Education, management, and administration	Case Study
2020	[34]	Cyber-Security Culture Awareness Framework	To evaluate organizational readiness from the cybersecurity domain perspective	Organization culture	No validation
2020	[24]	Effective corporatecommunication after cybersecurity incidents	The proposed framework can provide organizations with awareness into the kinds of actions that are essential after a cybersecurity incident	Business	Case study and interviews from experts
2019	[5]	Cybersecurity Awareness TrainingModel (CATRAM)	To provide training and awareness to the various organizations by considering the current cyberthreat landscape	Business	Case study
2018	[6]	Proactive Resilience Educational Framework (Proresilience EF)	Provide cybersecurity Education and Training in Hospitals	Healthcare	No validation
2019	[7]	Information security education training and awareness (SETA)	To provide sustainable behavioral change regarding cybersecurity by adopting behavior change wheel (BCW) framework	Healthcare	No validation
2020	[35]	Conceptual Model of Visual Analyticsfor Hands-on Cybersecurity Training	Using visual analytics to provide the sensemaking activities of users engaged in different phases ofthe training life cycle	Organization and education	Experiment by using KYPO cyber range (cloud-based platform)
2022	[36]	Factor model for online education during the COVID-19 pandemic	The proposed model offered recommendations for the government and universities for reducing the shortcomings regarding online education.	Education	Physical and online students survey
2022	[25]	Social education level on cybersecurity awareness and behavior	They measure the impact of the education level of the great societies to moderate the correlation in the middle of knowledge and attitude by using the knowledge–attitude–behavior (KAB) model.	Education	Questionnaire survey
2021	[27]	Cybersecurity awareness of employees	To find out the effect of cybersecurity awareness on worker behavior in Saudi Arabia environment.	Industry	Questionnaire survey
2022	[28]	Cybersecurity awareness (CSA) model	Proposed CSA model for railway organization in Thailand to detect the prominent factors.	Railway industry	Interview process from experts
2022	[37]	Cybersecurity Awareness Training Model	They established the significance of cybersecurity awareness training in incorporating with cyber-attacks and threats.	Organization	viCyber [38] Tool
2021	[39] (p. 19)	Cybersecurity awareness campaigns	To examine the usefulness of vulnerability revelation patterns on Twitter during the COVID-19 pandemic.	Organizations	Case study
2021	[30]	Cybersecurity awareness training	To investigate the efficiency of home management methods in decreasing cyber-attacks and threat incidents during online situations.	Organizations	Experiment
2022	[40]	Cybersecurity awareness measurement model	Constructed a method to assess the cybersecurity awareness level in water sector organizations	Water sector organization	Case study
2021	[41]	Conceptual model for cybersecurity governance	Developed a model to address the challenges related to cybersecurity governance.	Organizations	Survey
2021	[42]	Cybersecurity Awareness Framework	Recommended a conceptual Cybersecurity Awareness Framework to manage and direct the completion of methods to enhance cybersecurity awareness in the academic institution.	Academia	Survey
2021	[43]	Global Cyber Security Model	To guide cybersecurity manager to raise and conduct method for cybersecurity awareness among working employees	Organizations	Interview from experts

### Motivation

Research has been conducted on information security maturity and IT security maturity that focuses on the ability of the organizations to fulfill their security objectives [12,13,14,15], although these problems have been undervalued, misinterpreted, and not addressed in the manner that they should have been [12,13,44]. Different organizations still suffer security risks due to exploitation of security mistakes [45,46,47]. A challenge remains in integrating key cybersecurity practices in the form of a framework for employee awareness and training regarding various cyber-attacks and threats during the COVID-19 pandemic, and it is one that can be addressed through a CAT framework. Despite the significance of this issue, minimal research has been conducted to enhance cybersecurity awareness and training for online working employees after the COVID-19 pandemic.

The existing frameworks/models, as shown in Table 1, did not focus on cybersecurity awareness and training of their online-working-based employees. The current cybersecurity frameworks/models are generic and mostly address security at the software, hardware, and network levels and do not have defined practices and levels for cybersecurity awareness and training. Therefore, there is no framework to measure an organization’s online-working-based employees’ capability towards cybersecurity awareness and training.

This research study focuses on solving the challenges related to cybersecurity awareness and training. We propose a CAT framework for cybersecurity awareness and training that will help organizations to evaluate and measure their employees’ capability in cybersecurity. The proposed CAT framework will also assist different organizations to properly manage security-related issues and challenges in an effective and efficient manner to protect their assets and critical information.

## 3. Research Methodology

**Figure 1:**
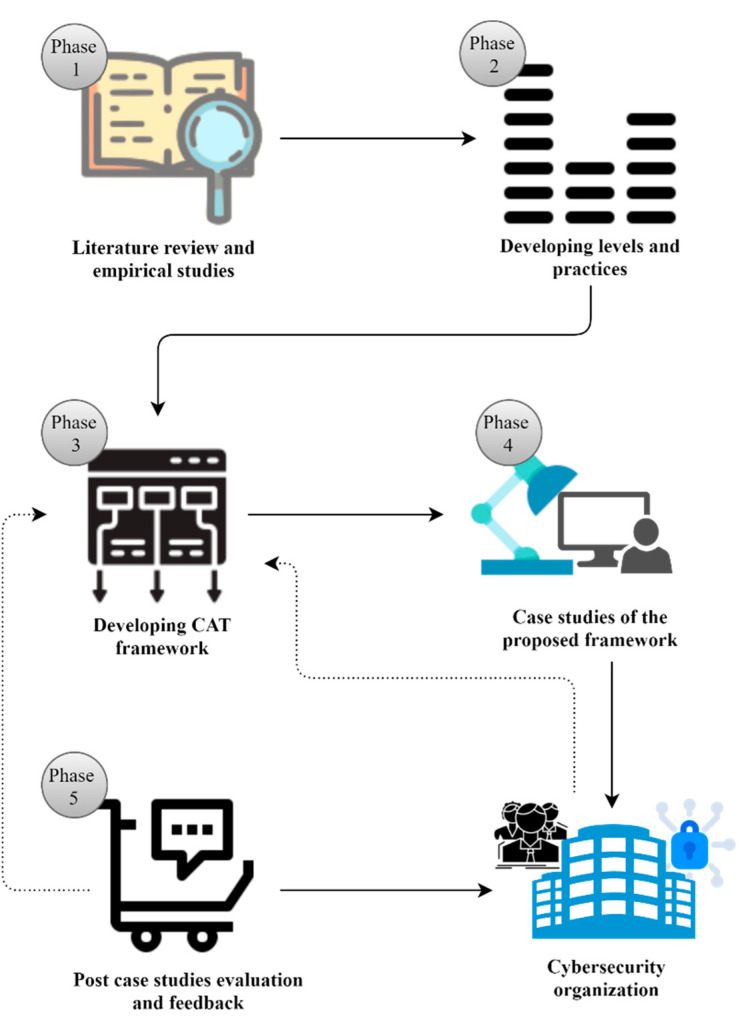
Proposed research methodology.

Research methodology is divided into the following five phases: literature review and empirical studies, developing CAT framework levels and practices, developing CAT framework, case studies, and, finally, post-case-study evaluation and feedback, as shown in Figure 1.

### 3.1. Literature Review and Empirical Studies

A comprehensive literature review was conducted in relevance to cybersecurity training and awareness. Empirical studies were also conducted to explore established frameworks/models in the cybersecurity domain. Comparison criteria such as name, reference, year, domain, and validation approaches were built to compare the identified cybersecurity awareness and training frameworks/models in different domains, such as healthcare, business, and education, as shown in Table 1.

### 3.2. Development CAT Framework Levels and Practices

We studied all the relevant empirical literature studies and already existing frameworks/models, including NIST (National Institute of Standards and Technology) “Building an information technology security awareness and training program” [8], to develop potential major CAT framework levels and key practices. Moreover, we interviewed security experts, engineers, testers, and developers to obtain knowledge about the development of CAT framework levels and practices.

### 3.3. Developing CAT Framework

An academic assessment was completed for building the proposed CAT framework related to cybersecurity training and awareness for online-working-based employees. This assessment facilitates us to build and verify the CAT framework and identify the weaknesses within it before applying the framework to the cybersecurity real-world environment for evaluation. After deep study and reviews, the proposed CAT framework was built free of flaws and errors and was ready for testing and evaluation in a real-world environment.

### 3.4. Case Studies of the Proposed CAT Framework

This phase includes sending the proposed developed CAT framework to the cybersecurity organizations for evaluation and measurement. It includes the extensive demonstration of the proposed CAT framework to determine whether it is suitable for application in a real-world environment, which will also lead towards further refinement of the proposed solution. The case study method was used to evaluate the CAT framework because it is a powerful evaluation tool that can provide useful real-world information [48]. A case study normally is the intersection of evidence from interviews, observation, questionnaires, and archives [49].

Next, a statistical analysis was performed to measure the proposed CAT framework and the conducted case studies. Statistical analysis is the most crucial phase because it assists with management of descriptive information; that is, numbers are assigned for every variable and their percentages that need to be described in the form of frequency graphs or tables. Frequencies are helpful in cases of comparison and contrasting among or across a group of variables by considering the four scales of data analysis, including nominal, ordinal interval, and ratio. In this research, the median is used for evaluation of our proposed CAT framework due to its ordinal scale nature.

### 3.5. Post-Case-Study Evaluation and Feedback

Post-case-study evaluations were completed to improve and enhance the developed CAT framework from the perspective of the real industrial environment. The framework was evaluated based on three main aspects: ease of use, CAT framework structure, and user satisfaction in order to improve it and make it more feasible for the organizations who want to use it.

## 4. Cybersecurity Awareness and Training (CAT) Framework

### 4.1. The Development Process of the CAT Framework

The CAT framework is primarily founded on the structural concept of the well-known NIST “Building an information technology security awareness and training program” [8]. We developed the CAT framework by introducing AI concepts consisting of machine learning, natural language processing, and expert systems to make our proposed model self-adaptive and smart. Further, we built an adaptive-based knowledge measurement module by including threshold value points. Likewise, we divided the CAT framework into three major levels: beginner (awareness), medium (training), and advanced (practical and assessment). Each level is further divided into its key practices. The levels and key practices of the CAT framework were established from the conducted literature review, relevant empirical studies, and from the NIST “Building an information technology security awareness and training program” [8].

**Figure 2:**
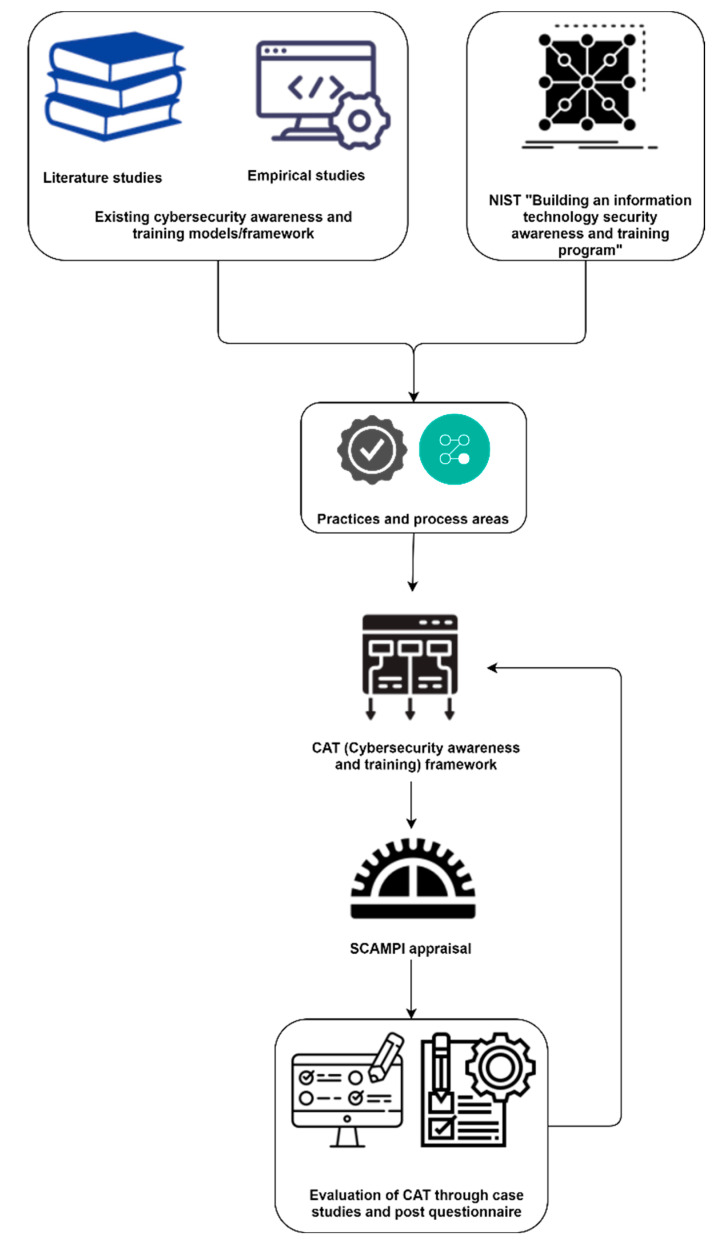
Complete development process of CAT.

The authors repeatedly reviewed the levels with practices and placed them in their proper position in each level of the CAT framework. Before the real-world environment case studies evaluation, a review of the CAT framework was performed many times by the researchers in an iterative manner. The complete flow of the development process of the CAT framework is shown in Figure 2.

### 4.2. Structure of the CAT Framework

The structure of the CAT framework was implemented mainly from NIST (National Institute of Standards and Technology) “Building an information technology security awareness and training program”. NIST provides guidance on creating an efficient IT security program and maintaining the requirements stipulated in the Federal Information Security Management Act (FISMA) [50] and the Office of Management and Budget (OMB) [51]. Moreover, NIST states that a robust IT security program cannot be put in place when deprived of the important consideration given to “training agency IT users on security policy, procedures, and techniques, as well as the various management, operational, and technical controls necessary and available to secure IT resources”. Failure to provide attention to the domain of security training puts an organization at massive risk because “security of agency resources is as much a human issue as it is a technology issue”. Finally, NIST divides its documents into four major categories: (1) Awareness and Training Program Design, (2) Awareness and Training Material Development, (3) Program Implementation, and (4) post-Implementation. We also took key information from the various literature studies and from empirical studies to add to the proposed CAT framework. However, none of the frameworks or models have focused specifically on a cybersecurity awareness and training program for online-working-based employees during COVID-19 and onwards, hence the need for a new framework for this modern era.

**Figure 3:**
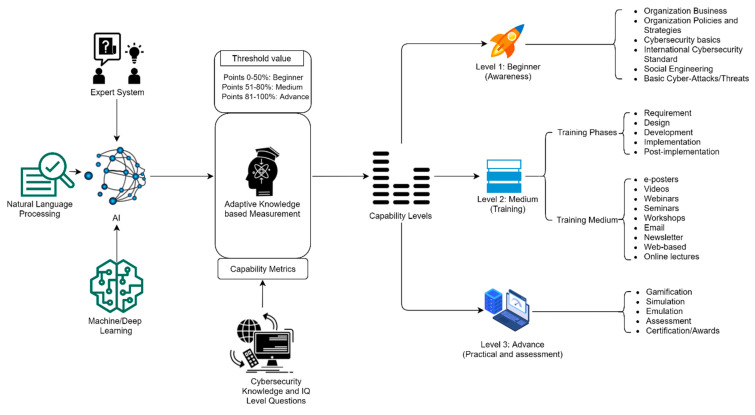
Entire structure of the CAT framework.

We developed the structure of the proposed CAT framework into three main modules, namely: (1) artificial intelligence (AI), (2) adaptive-knowledge-based measurement, and (3) capability levels. Figure 3 shows a detailed overview of the proposed CAT model.

### 4.3. AI Module

This module is further divided into three main components: machine learning (ML), natural language processing, and expert systems. When applying ML and AI techniques, we will consider and assess security for the proposed framework to avoid any compromise or generation of security risks. According to AWS security guidelines for ML, the basic security measures are data poisoning, membership inference, and model inversion [52]. Implementing these AWS basic features can help to prevent security breaches against AI and ML techniques.

#### 4.3.1. Machine/Deep Learning

Machine and deep learning algorithms are applied for modeling and developing students’ knowledge [53]. Different machine learning and deep learning techniques are used for self-adaptive testing mechanisms. Similarly, these techniques are also applied for developing cybersecurity awareness and training and testing modules to measure trainees’ ability based on their knowledge. Based on the historical data, various machine learning and deep learning techniques are used to predict the improvement and optimization of the training program [54].

#### 4.3.2. Natural Language Processing

Natural language processing is an artificial intelligence technique that is closely used for automated scoring self-adaptive testing systems [55]. The present work includes information gathered from the related text and speech data from natural language processing used by the educational technology and organizations, which addresses the requirements of teachers and students [56]. By using natural language processing, we can automate and improve the cybersecurity awareness and training framework to enhance the automated scoring self-adaptive testing system.

#### 4.3.3. Expert Systems

Within the domain of artificial intelligence, an expert system looks like a computer system mimicking the decision-making ability of a human expert. It is designed to resolve complicated problems by reasoning through bodies of knowledge, characterized primarily as if–then rules. Expert systems are mainly used for educational decision-making [57]. Similarly, Hwang et al. [58] used the expert system approach by bringing into account both the affective and cognitive condition of the individual learner. By applying an expert system to the cybersecurity awareness and training program, we can improve the learning process of the trainee.

### 4.4. Adaptive-Knowledge-Based Measurement Module

This module has a capability metric for the proceeding three levels (beginner, medium, advanced). The threshold values specified for each level are beginner: 0–50%; medium: 51–80%; and advanced: 81–100%. This module includes cybersecurity knowledge and IQ-level questions written and quantified by the organization according to their domain and nature of system security for their online workforces.

### 4.5. Capability Levels Module

Capability levels are further divided into the following:

#### 4.5.1. Beginner

This level consists of basic awareness regarding the organization’s cybersecurity. Following are the required key components of this level:

Organization business: This is the working domain of an organization, such as healthcare, finance, transportation, information technology, etc. The organization needs to define its domain and business processes explicitly for its workforces.

Organization policies and strategies: Achievement of an organization is directly associated with how the employees and overall administration perceive the objectives to be realized, and the methods were developed to attain their goals. A strategy is an act that the managers and directors undertake to achieve their organizational goals. A policy is a set of rules and guidelines prepared by the organization for reasonable decision-making. Organizations must introduce their strategies and policies clearly to the employees and other relevant people in the organization to achieve the desired goals and marketing targets.

Cybersecurity basics: This component includes basic knowledge of cybersecurity. The organization must make their employees and administration aware of the basic cybersecurity knowledge. Cybersecurity is established on three important concepts called “The CIA Triad”, which signifies the confidentiality, integrity, and availability that should be defined and conveyed to their employees. Other necessary terms need to be defined, such as: “asset management and identification, risk management, access management, threat management, security controls, disaster recovery and business continuity, incident management, security education, training, and awareness”.

International cybersecurity standard: Cybersecurity standards are collected works of the best practices established by the domain experts to defend organizations from cyber-attacks and threats. The proposed standards and frameworks are usually appropriate for all organizations, irrespective of their size and scope. The organization needs their employees and administration to be aware of the cybersecurity international standards to protect the organization’s assets from cyber-attacks and threats. The major and most well-known international cybersecurity standards include but are not limited to the following: DFARS (Defense Federal Acquisition Regulation Supplement) [59], Federal Information Security Management Act (FISMA) [60], Health Insurance Portability and Accountability Act (HIPAA) [61], ISO 22301 [62], ISO/IEC 27001, ISO/IEC 27031 [63], NIST Cybersecurity Framework (CSF) [64].

Social engineering: Social engineering (SE) is a method frequently used by hackers and cybercriminals to trick people into giving them access to a system by breaking the security practices and standards. The major social engineering cyber-attacks are accomplished through social media platforms, such as Facebook, Twitter, Instagram, Snapchat, and YouTube [4,65,66,67]. Those platforms appeal to hackers because of the public’s deficiency in awareness. The hackers use different social engineering techniques, such as phishing, smashing, vishing, pretexting, dump diving, extortion, etc. The employees of any organization need to be aware of social engineering techniques and their employed mechanism of cyber-attacks and threats.

Basic cyber-attacks and threats: A cyber-attack is a malicious act that seeks to damage data, steal data, or disrupt digital life in general. Organizations are required to train their employees and administration regarding the most common cyber-attacks and threats and to teach them how to manage and mitigate these kinds of cyber-attacks and threats. For example, cyber-attacks and threats consist of “computer viruses, data breaches, malware, spyware, phishing attacks, ransomware, zero-day exploits, advanced persistent threats, trojans, wiper attacks, intellectual property theft, theft of money, data manipulation, data destruction, man-in-the-middle attack, drive-by downloads, malvertising, rogue software, unpatched software, data center disrupted by a natural disaster, Denial of Service (DoS) attacks and other attack vectors”.

#### 4.5.2. Medium

The medium level includes the following training phases and training platforms.

Training phases: The training phases are conducted to train and bring awareness of cybersecurity challenges, attacks, and threats to the organization’s employees. These phases are organized in a systematic structure that consists of the training requirement, design, development, implementation, and post-implementation.

Requirement: Cybersecurity awareness and training requires continuing education that provides employees significant information and an examination of their cybersecurity training and awareness by including all facets of data security and regulatory compliance. Humans are the weakest link in cybersecurity [65,68], and we need to be trained and made aware of the current cybersecurity challenges and trends. This phase is in place to determine the employee or group of employees that need training and awareness. Moreover, by selecting the appropriate medium of training, a company can organize employees’ schedule and benefits to improve learning. Furthermore, cybersecurity topics to be covered are based on the employee’s background cost of training, the scope of the training, strategies, and policies for conducting training, roles and responsibilities, training tutors, and organizers.

Design: This phase must be designed in a manner that keeps the organization’s mission and goals in mind. This phase is significant and supports the business’s needs and is related to the organization’s culture and IT infrastructure. The training design phase defined the overall training model from start to end by keeping all the essential components in mind, such as training all stockholders, seekers, and givers; timeframe; the relevant topics on cybersecurity for training; and presenting clear guidelines.

Development: This is the development and realistic structure of all the components mentioned in the requirement and design phase of the training and awareness process. Moreover, in this step, the components are refined by adding or removing certain components to achieve quality and training satisfaction.

Implementation: This is the actual implementation of the training and awareness programs for the specified online-working-based workforces, keeping in mind the supportive human and non-human resources, understandability level, and appropriate way of delivering the training to the trainee.

Post-implementation: This phase is intended to ensure that the trainees advance from beginner by conducting regular assessments, regular interactions, ensure professional growth, knowledge testing, compile their results, and support improvement. This ensures the trainee achieved the necessary competent knowledge according to their role in the organization.

Training medium: This comprises the medium for conveying the training. The use of training medium depends on the organization requirements, domain, and structure. Following are the training mediums:

- E-posters
- Videos
- Webinars
- Seminars
- Workshops
- Email
- Newsletters
- Web-based
- Online lectures.

#### 4.5.3. Advance

The advanced level includes the practical assessment and normally comes after completing the previous levels. The advanced level includes the following practical and assessment strategies.

*Gamification:* Researchers and practitioners [69,70,71,72] have been developing games for cybersecurity training because of user interest and interactive interface, offering some gaming challenges and a sharper level of thinking. Learning through gaming establishes an immersive, learner-focused experience. It is an efficient way to enact cybersecurity awareness training with a practical skill achievement for trainees from various qualifications and environment backgrounds. Several games that have already been developed for cybersecurity training are cybersecurity lab [73], zero threats [74], keep tradition secure [75], and game of threats [76,77].

*Simulation/Emulation:* A simulation/emulation is an animated model that mimics the operation and processes of the proposed cybersecurity system, such as cybersecurity attacks, threats, incidence response, management, and mitigation. Simulations are good for trainees because the learners can damage the actual resources at their initial level of training without presenting the actual environment. Simulation training is possible to conduct online and is less expensive than in-person training. Researchers and practitioners have already worked to provide cybersecurity training simulators [78,79,80,81]. Joseph Mayes from the Software Engineering Institute [82] mentioned that some open source tools for creating cyber simulators are TopoMojo, GreyBox, vTunnel, GHOSTS, and TopGen.

*Assessment:* The assessment ensures the trainee’s professional growth, knowledge testing, compiles their results, and supports improvement. The assessment checks that the trainee achieved the necessary knowledge according to their roles in the organization.

*Certificates/awards:* Certificates or awards are given to the employee after successful completion of the cybersecurity training and awareness program.

### 4.6. Capability Levels and Scoring Criteria of the CAT Framework

The CAT framework consists of three capability levels, which were adopted mainly from the NIST (National Institute of Standards and Technology) “Building an information technology security awareness and training program” and the relevant empirical studies. Following are the capability levels scoring criteria:

- Beginner

This capability level only includes a basic awareness of cybersecurity. The qualitative point score for this capability level is between 0% and 50%.

- Medium

This capability level includes the cybersecurity program training. The qualitative point score for this capability level is greater than 50% and less than or equal to 80%.

- Advanced

This capability level includes the comprehensive reviews and the practical and final assessment of the cybersecurity awareness and training program. The qualitative point score for this capability level is greater than 80% and less than or equal to 100%.

The capability levels’ percentage range values can be modified by the organization according to their priorities without affecting the capability levels and the overall measurement of the proposed framework.

The CAT framework adopted the range values from the IBM (RUP) process area, and the exclusion of percentages in the IBM process area was replaced by corresponding number values beginning at 1, equal to the beginner capability level, 2 for medium, and, finally, 3 for advanced. Then, multiply the percentage values with the highest-level scale in our circumstance, which is 3, to achieve the equivalent value for each capability level. For example, 0.50 × 3 is equal to 1.5, 0.80 × 3 is equal to 2.4, and, finally, 0.100 × 3 is equal to 3. The median range has been calculated and adopted from the study conducted by Grundmann [60]. We used SCAMPI [61] for the assessment of our practice’s capability as it is commonly used to support quality scoring benchmarks. SCAMPI was utilized as an appraisal concept and structure in order to evaluate the capability of every practice and the CAT framework. Table 2 shows the value range in detail with the capability levels according to IBM (RUP).

## 5. Evaluation of CAT

Two case studies were conducted to evaluate the CAT framework in a real-world environment within a reputed cybersecurity international organization. Cybersecurity experts and engineers along with their teams from the selected organizations agreed to contribute to the case studies. They showed a desire to measure the capability of the practices that are defined in the proposed CAT framework in their organizations. Our research team provided all the applicable complete documentation and guidelines for efficiently conducting the case studies to avoid any biases and mistakes. Our research team provided participants with an Excel spreadsheet containing the levels and practices that were developed in the proposed CAT framework.

The conducted case studies of two selected organizations are mentioned in Section 5.1.1 and Section 5.1.2. The main purpose of the provided Excel sheets was to take the opinions and suggestions in digits (assigned numbers) in the case studies and post-case-study surveys from the security experts. Through scripting, the Excel sheet can automatically calculate the median of the input opinions of the experts. The high-rank security experts of the specific organizations conducted case studies with their respective colleagues in a one-month time frame. Examples of the Excel sheet are shown in Appendix A.

We requested that the participants assess every practice of the CAT framework for the mentioned capability levels, assigning point values per the following:

1. If the organization did not apply the practice of the CAT framework.
2. If the organization partially applied the practice of the CAT framework.
3. If the organization completely performed applied the practice of the CAT framework.

### 5.1. Case Study

Case study [48] is a research approach that is commonly used by various fields, such as life sciences, social sciences, and different engineering disciplines, to validate the intended case or experiment in a practical environment. It is established on formal research, and most researchers use this method with research to be published in journals and conferences. A case study contains both qualitative and quantitative methods and is mainly used for validation purposes of theoretical and conceptual models and frameworks. A case study is typically the intersection of evidence from collection methods, such as interviews, observations, questionnaires, and archives [49].

#### 5.1.1. Organization I

According to the demographics of organization I, it is an international private cybersecurity organization located in Pakistan, and over 60 employees are working in this organization. It delivers cybersecurity solutions, penetration testing, and consultations. The working domain in which this organization provides its services is safety-critical systems, business systems, communications, and real-time systems. The case study was conducted at the main branch of this organization. The responses for the case study were submitted by the senior cybersecurity team lead, who has more than 8 years of experience. The following levels of results were achieved from organization I.

*Beginner*

The median of this level of evaluation is 2, which is the awareness level of the CAT framework. This cybersecurity organization has not reached the highest capability level. If this organization wants to jump into the highest level of capability in this phase, then they must follow and implement the other practices in the beginner and medium levels. Furthermore, the organization must work on all practices except organizational policies and strategies and cybersecurity basic knowledge, which is already at the advanced capability level.

*Medium*

The median of this evaluation level is 2, which is the training capability level of the CAT framework. Similarly, this cybersecurity organization has not reached the highest capability level. If this organization wants to grow to the highest level of capability in cybersecurity awareness and training, then they must follow and implement the other practices that are marked as the beginner and medium levels in the case study of the proposed CAT framework. This level needs more effort with the practices in the beginner and medium levels, especially the training phases practices. Efforts are needed in all practices except email and web-based training platforms because they have achieved advanced capability level in those practices.

*Advanced*

The median of this evaluation level is 2, which is the practical and assessment capability level of the CAT framework, as in the other levels. If this organization wants to grow towards the highest level of capability, then it must follow and implement the other practices that are marked with beginner and medium capability levels. The practical and assessment are almost in the beginner and medium capability levels. They only had two practices at the highest capability level, which shows a clear indication and needs more work to achieve the advanced capability level.

The overall outcomes of the case study evaluation of organization I are quite near the advanced level of the CAT framework. They have achieved the medium capability level. Then again, the organization still needs to implement more practices of the overall three levels of the CAT framework because security is the most significant factor of any organization in protecting their critical assets and information. Some practices and certain levels still need to be advanced by reaching the advanced level of the CAT framework.

#### 5.1.2. Organization II

Organization II is an international private cybersecurity organization. Its offices are located in the three major cities of Pakistan. It is an independent organization providing solutions and training in the cybersecurity domain. The case study was conducted at the main branch. More than 100 employees work in this organization, and 400-plus clients are registered worldwide. Similarly, they have trained over 1500 information security professionals. They deliver both national and multi-national services. They work in the security management, maturity assessment, security consultancy, security awareness, and assessment domains. The responses to the case study were provided by the senior information security officer, who has more than 10 years of experience. This organization case study gives us more confidence in the proposed CAT framework because it provides awareness and training programs to cybersecurity professionals.

*Beginner*

The median of this evaluation level is 2, which is the awareness capability level of the CAT framework. This software organization has not reached the highest capability level. In this level, only one practice achieved the beginner capability level, which is social engineering, and one practice attained the advanced level, which is cybersecurity basics. All the other practices were achieved at the medium capability.

*Medium*

The median of this evaluation level is also 2, which is the training capability level of the proposed CAT framework. The training development and email and web-based training platforms had a quite good evaluation and almost reached the highest advanced capability level. However, five practices had the lowest beginner capability level, specifically online lecture training platform and requirement, design, and post-implementation of training. The other remaining practices are at the medium capability level, so more effort is required to achieve the advanced capability levels.

*Advanced*

As with the other two levels, the median of this evaluation level is also 2, which is the medium capability level of the CAT framework. Two practices, simulation and emulation, fell at the beginner level, and one practice, known as assessment, received the medium level. However, this organization is more focused on gamification and certification/wards. If this organization wants to obtain the highest level of capability in this level, then they must follow and implement the other practices that are marked with beginner and medium capability levels.

The overall outcomes of the case study evaluation for organization II are relatively near the advanced level of the proposed CAT framework. However, the organization still needs to implement more practices of the overall three levels of the CAT framework. This organization is quite good compared to organization I because it is conducting and providing more focus to training and certification of cybersecurity professionals.

## 6. The Post-Case-Study Feedback Questionnaire from Case Studies Implemented by Organizations

After the completion of the case studies from both organizations, the contributors were invited to complete a post-case-study questionnaire to offer their opinion about the CAT framework structure, user satisfaction, and ease of use. The responses about the CAT framework were carefully assessed and evaluated for the goal of improvement. The tables and success criteria for each table were adopted from the published study by Mufti et al. [83].

In the first phase, we requested that the organizations evaluate the ease of use of the CAT framework, and both organizations I and II agreed and strongly agreed that the CAT framework is easy to comprehend and use. The contributors of the questionnaire have grasped the background and had important knowledge regarding cybersecurity, practices, and processes. Table 3 includes the ease-of-use evaluation.

In the second phase, we asked the participants about the user satisfaction of the CAT framework. The user satisfaction was measured and evaluated based on the CAT framework outcomes. Both organization I and organization II agreed and strongly agreed about the satisfaction of the CAT framework, and they suggested the CAT framework to other organizations as well. Table 4 shows the user satisfaction evaluation.

In the third phase, we asked the participants about the structure of the CAT framework. Both organizations evaluated and measured the structure of the CAT framework. They provided us very positive reactions regarding the simplicity of the structure and CAT framework organization. Moreover, they most strongly agreed on the proposed levels and practices, and especially the placement position of the practices in each level of the CAT framework. Table 5 shows the MMSST structure evaluation.

In the final phase, we requested that the questionnaire contributors provide successful and considerable suggestions to improve the proposed CAT framework. However, we did not receive any potential comments or suggestions from either organization I or II, which clearly revealed that the proposed CAT framework could assess the employees’ awareness and training on the perspective of cybersecurity. The post-case-study comments from both organizations I and II are shown in Table 6.

## 7. Study Limitations

The case study request was sent to many organizations, but most of them were reluctant to complete the case study and provide us their responses. The cause may be hesitation regarding the organization’s reputation and privacy. However, we explicitly stated in the proposed case study instructions that the information would be held in the strictest confidence. For the proposed CAT framework generalization, we are seeking a few more reputed cybersecurity-relevant organizations around the globe to contribute to the proposed case studies. The assessment results of the CAT framework were obtained from two international and reputed cybersecurity organizations.

Another potential limitation is related to the literature review process regarding that some research papers may have been overlooked. However, we believe that our results cover most of the appropriate published research studies. Further, it is probable that subjective decisions had an impact regarding collection of major research studies in the information mining stages because some of the major research studies did not utilize organized abstracts, concise descriptions in the discussion section, and clear contributions of their research work. However, to lessen this constraint, the authors endeavored to carry out the literature review process as well as possible in order to obtain rich information.

## 8. Conclusions and Future Work

Cybersecurity is currently an emergent research area due to a variety of cyber-attacks that precede massive financial loss to organizations’ reputations. Researchers and practitioners now consider cybersecurity critical due to different cyber threats and attacks on various organizations during the COVID-19 pandemic. Organizations are attempting to protect essential assets and information from different kinds of hackers and threat actors. The main objective of this research was to develop a CAT framework to assist different domain organizations in protecting their key assets and information from those threats. Organizations need to implement the proposed CAT framework to provide awareness to and train their employees regarding the recent social-engineering-based cyber-attacks and threats. The proposed CAT framework will help organizations to identify their cybersecurity-relevant weaknesses in their systems and measure their employees’ capability toward cyber threats, attacks, and incidence management. Moreover, implementing the categorized practices of each level of the CAT framework will improve the organization’s security infrastructure. A comprehensive literature review was conducted to point out the potential practices of the proposed framework from the perspectives of experienced researchers in the domain of cybersecurity.

The structure of the proposed CAT framework includes three main levels: beginner (awareness), medium (training), and advanced (practical and assessment). Similarly, we identified a total of 25 most important practices for achieving the developed levels. A case study was established and conducted for evaluation of the proposed CAT framework within cybersecurity organizations for validation and further improvement purposes. Case studies were implemented in two organizations only at the initial stage, and the results were achieved. The obtained results gave us more confidence in our proposed CAT framework because both organizations gained a medium level. However, they still need to implement more practices from the overall three levels of the CAT framework because security is the most crucial factor in protecting their organizational core assets and information.

In future work, we will compare our proposed CAT framework with other cybersecurity training and awareness frameworks that are already in the operational stage to obtain a more transparent view of the practical implementation by the organizations. Moreover, we will propose an automated software tool in combination with AI for the proposed CAT framework. The software tool will support organizations in easy implementation and measurement of their employees’ cybersecurity awareness and training capability in real time.

## Figures and Tables

**Table 2:** Scoring criteria of the CAT framework.

No.	Range Value in % by IBM	Range of Median Value for CAT	Capability Level
1	0–50%	If 0 < Med <= 1.5	Beginner
2	51–80%	If 1.5 < Med <= 2.4	Medium
3	81–100%	If 2.4 < Med <= 3	Advance

**Table 3:** CAT framework ease of use evaluation from both organization I and Organization II.

No.	CAT Framework Ease of Use	Organizations’ Viewpoint (*n* = 2)							
		Strongly Agree and Agree			Strongly Disagree and Disagree			Neutral	
		Strongly Agree	Agree	Percentage of Strongly Agree or Agree	Strongly Disagree	Disagree	Percentage of Strongly Disagree or Disagree	Neutral	Percentage of Neutral
1	CAT framework demonstration is straightforward to understand and learn.	2	0	100	0	0	0	0	0
2	Basic knowledge applicable to cybersecurity is essential to be able to make use of the CAT framework.	2	0	100	0	0	0	0	0
3	The practices for processes are important to understand and learn for each CAT framework level.	0	2	100	0	0	0	0	0
4	It is crucial to use the CAT framework to measure an organization employee’s capability for CAT framework levels and practices.	2	0	100	0	0	0	0	0
5	Awareness and training are needed to enable the use of CAT framework.	2	0	100	0	0	0	0	0

**Table 4:** CAT framework user satisfaction evaluation from both organization I and organization II.

No.	CAT Framework User Satisfaction	Organizations’ Perception (*n* = 2)							
		Strongly Agree and Agree			Strongly Disagree and Disagree			Neutral	
		Strongly Agree	Agree	Percentage of Strongly Agree or Agree	Strongly Disagree	Disagree	Percentage of Strongly Disagree or Disagree	Neutral	Percentage of Neutral
1	CAT framework can be applied to highest number of organizations.	2	0	100	0	0	0	0	0
2	Every Single practice of CAT framework is easy and useable.	0	2	100	0	0	0	0	0
3	CAT framework can identify the weak and strong spots in organizations employee’s relation to CAT framework levels and their practices which they perform.	0	2	100	0	0	0	0	0
4	The use of CAT framework would improve cybersecurity awareness and training.	2	0	100	0	0	0	0	0
5	If the CAT framework were available in my organization, I believe to use it.	0	2	100	0	0	0	0	0
6	I agree with the cybersecurity levels and practices documented by CAT framework.	0	2	100	0	0	0	0	0
7	Utilizing the CAT framework as a practical software tool is essential to cybersecurity training and awareness for measuring the organization’s employee capability.	2	0	100	0	0	0	0	0

**Table 5:** CAT framework structure evaluation from both organization I and organization II.

No.	CAT Framework Structure	Organizations’ Perception (*n* = 2)							
		Strongly Agree and Agree			Strongly Disagree and Disagree			Neutral	
		Strongly Agree	Agree	Percentage of Strongly Agree or Agree	Strongly Disagree	Disagree	Percentage of Strongly Disagree or Disagree	Neutral	Percentage of Neutral
1	Every level of the CAT framework is understandable and intends no further clarification for acceptable use.	0	2	100	0	0	0	0	0
2	Every level of the CAT framework is satisfactory and applicable to the awareness and training of the organization’s employees.	2	0	100	0	0	0	0	0
3	CAT framework can be applied excellently to identify cybersecurity weaknesses of the organization with an aim to grow up organization’s employee’s capability for awareness and training of cybersecurity.	0	2	100	0	0	0	0	0
4	The distribution of cybersecurity practices among different levels (e.g., Beginner, medium and Advance) is useful.	2	0	100	0	0	0	0	0
5	The three levels of the CAT framework are helpful.	2	0	100	0	0	0	0	0

**Table 6:** CAT framework post-case-study responses from both organization I and organization II.

Question	Response		
	Organization I	Organization II	Outcome
Do you think there is a missing practice that needs to be added to the CAT framework? Please provide reasons for your answer.	Revise the practices again, may be some practices are missed	No	Encouraging
Do you have any recommendations to make better the proposed CAT framework?	No	Provide full documentation of the practices that guides novice workers in the area having no knowledge and experience in cybersecurity.	Encouraging
Any comments or suggestions expected on the assessment method of the CAT framework?	Would be better to provide an automated tool in the future to make it easy for the organization to assess their employees/workers capability.	No	Encouraging
Have any CAT framework level practices been inaccurately categorized?	No	No	Very Positive

## Data Availability

The data are available upon request via corresponding author email.

## Appendix A

**Table A1:** Case Study Excel Sheet Example of an Anonymous Organization.

**Please Fill Each Rows of**Table 1, Table 2 and Table 3 **with One of These Values Except the Yellow Colour Row**				
**1**	If this level is **Beginner **applied for Awareness capability.			
**2**	If this level is **Medium**applied for training capability.			
**3**	If this level is **Advance**applied for practical and assessment capability.			
	Range value in % by IBM	Range of Average Value for CAT framework	Capability Level	
1	0–50%	If 0 <Avg. <= 1.5	Beginner	
2	51–80%	If 1.5 <Avg. <= 2.4	Medium	
3	81–100%	If 2.4 <Avg. <= 3	Advance	
**ID**	**Level and Practices**	**Awareness Level**		
		**Beginner**	**Medium**	**Advance**
**Level 1**	**Awareness**	**1**	**2**	**3**
**P1.1**	Organization business			3
**P1.2**	Organization polices and strategies			3
**P1.3**	Cybersecurity basics		2	
**P1.4**	International cybersecurity standards		2	
**P1.5**	Social engineering		2	
**P1.6**	Basic cyber-attacks and threats		2	
		**2**		
**The Outcome of SCAMPI Appraisal for CAT framework awareness level practices which the organization covers is:**		**Median**	**Appraisal of Organization Using SCAMPI**	
		**2**	**Medium**	
**ID**	**Level and Practices**	**Training Level**		
		**Understanding**	**Improvement**	**Advance**
**level 2**	**Training**	**1**	**2**	**3**
	** Training phases**			
P2.1	Requirements		2	
P2.2	Design the peer review	1		
P2.3	Development		2	
P2.4	Implementation		2	
P2.5	Post-implementation	1		
	** Training Medium**			
P 2.6	E-poster			3
P 2.7	Videos			3
P 2.8	Webinars		2	
P 2.9	Seminars		2	
P 2.10	Workshops	1		
P 2.11	Email		2	
P 2.12	Newsletter			3
P 2.13	Web-based		2	
P 2.14	Online lectures		2	
		**2**		
**The Outcome of SCAMPI Appraisal for CAT framework training level practices which the organization covers is:**		**Median**	**Appraisal of Organization Using SCAMPI**	
		**2**	**Medium**	
**ID**	**Level and Practices**	**Practical and Assessment Level**		
		**Understanding**	**Improvement**	**Advance**
**Level 3**	**Practical and assessment**	**1**	**2**	**3**
P 3.1	Gamification		2	
P 3.2	Simulation		2	
P 3.3	Emulation	1		
P 3.4	Assessment		2	
P 3.5	Certification/Awards		2	
		**2**		
**The Outcome of SCAMPI Apprisal for CAT framework practical and assessment level practices which the organazation covers is:**		**Median**	**Appraisal of Organization Using SCAMPI**	
		**2**	**Medium**	
**Overall Summary Report of CAT Framework within Organization Case Study:**				
**No.**	**Three Levels of CAT Framework**	**Median**	**Appraisal of Cybersecurity Organization Using SCAMPI**	
**1**	** Awareness**	**2**	Medium	
**2**	** Training**	**2**	Medium	
**3**	** Practical and Assessment**	**2**	Medium	
